# An Aberrant Splice Acceptor Site Due to a Novel Intronic Nucleotide Substitution in *MSX1* Gene Is the Cause of Congenital Tooth Agenesis in a Japanese Family

**DOI:** 10.1371/journal.pone.0128227

**Published:** 2015-06-01

**Authors:** Tadashi Tatematsu, Masashi Kimura, Mitsuko Nakashima, Junichiro Machida, Seishi Yamaguchi, Akio Shibata, Hiroki Goto, Atsuo Nakayama, Yujiro Higashi, Hitoshi Miyachi, Kazuo Shimozato, Naomichi Matsumoto, Yoshihito Tokita

**Affiliations:** 1 Department of Maxillofacial Surgery, Aichi-Gakuin University School of Dentistry, Nagoya, Aichi, Japan; 2 Department of Dentistry Oral and Maxillofacial Surgery, Ogaki Municipal Hospital, Ogaki, Gifu, Japan; 3 Department of Human Genetics, Yokohama City University Graduate School of Medicine, Yokohama, Kanagawa, Japan; 4 Department of Oral and Maxillofacial Surgery, Toyota Memorial Hospital, Toyota, Aichi, Japan; 5 Department of Dentistry and Oral Surgery, Aichi Children’s Health and Medical Center, Obu, Aichi, Japan; 6 Department of Embryology, Institute for Developmental Research, Aichi-Human Service Center, Kasugai, Aichi, Japan; 7 Department of Perinatology, Institute for Developmental Research, Aichi-Human Service Center, Kasugai, Aichi, Japan; International Centre for Genetic Engineering and Biotechnology, ITALY

## Abstract

Congenital tooth agenesis is caused by mutations in the *MSX1*, *PAX9*, *WNT10A*, or *AXIN2* genes. Here, we report a Japanese family with nonsyndromic tooth agenesis caused by a novel nucleotide substitution in the intronic region between exons 1 and 2 of the *MSX1* gene. Because the mutation is located 9 bp before exon 2 (c.452-9G>A), we speculated that the nucleotide substitution would generate an abnormal splice site. Using cDNA analysis of an immortalized patient blood cell, we confirmed that an additional 7-nucleotide sequence was inserted at the splice junction between exons 1 and 2 (c.451_452insCCCTCAG). The consequent frameshift generated a homeodomain-truncated MSX1 (p.R151fsX20). We then studied the subcellular localization of truncated MSX1 protein in COS cells, and observed that it had a whole cell distribution more than a nuclear localization, compared to that of wild-type protein. This result suggests a deletion of the nuclear localization signal, which is mapped to the MSX1 homeodomain. These results indicate that this novel intronic nucleotide substitution is the cause of tooth agenesis in this family. To date, most MSX1 variants isolated from patients with tooth agenesis involve single amino acid substitutions in the highly conserved homeodomain or deletion mutants caused by frameshift or nonsense mutations. We here report a rare case of an intronic mutation of the *MSX1* gene responsible for human tooth agenesis. In addition, the missing tooth patterns were slightly but significantly different between an affected monozygotic twin pair of this family, showing that epigenetic or environmental factors also affect the phenotypic variations of missing teeth among patients with nonsyndromic tooth agenesis caused by an *MSX1* haploinsufficiency.

## Introduction

Although nonsyndromic tooth agenesis is one of the most common developmental anomalies in humans, its cause is largely unknown [1]. Tooth agenesis is classified into two subtypes according to the number of missing teeth: hypodontia (one to five missing teeth, excluding the third molar) and oligodontia (six or more missing teeth, excluding the third molar). Recently, we reported that the prevalences of these two subtypes in the Japanese population are 6.8% (hypodontia) and 0.1% (oligodontia) and that the sibling recurrence risks are 25.0% and 43.8%, respectively, suggesting that the severe phenotype, oligodontia, may be mostly transmitted in a dominant fashion [2].

Multiple congenitally missing teeth have been associated with mutations in genes such as *MSX1*, *EDA*, *AXIN2*, *PAX9*, *WNT10A*, *EDAR*, and *EDARADD* [3–12]. The human *MSX1* gene is mapped to chromosome 4 and contains two exons of 704 bp and 1236 bp, which are separated by a 2332-bp intron. *MSX1* expression appears during early tooth development, and mutations in this gene are involved in human isolated tooth agenesis [6, 13–23], tooth agenesis with nail dysplasia [24], and tooth agenesis with cleft lip and palate [25–27].

In our current study, we used whole-exome sequencing (WES) to investigate a three-generation Japanese family with tooth agenesis and attempt to identify the gene mutations that had caused this condition. We identified a novel single nucleotide substitution in the *MSX1* intronic region and revealed that it leads to an aberrant splice site at 7bp before original splice acceptor site of exon 2, and results in a C-terminal truncated gene product.

## Patients and Methods

### Family and pedigree analysis

A 28-year-old Japanese woman was referred to the Maxillofacial Surgery, Aichi-Gakuin University School of Dentistry. A panoramic radiograph was taken to verify the exact number of missing teeth. Her family members, including monozygotic twin brothers, were subsequently recruited. This study was approved by the Committee on the Ethics of Human Experimentation, Aichi-Gakuin University (Approval number: #58), and the Institute for Developmental Research (Approval number #13–07). A blood or hair sample was obtained from the participants with written informed consent.

### Mutational analysis with WES

**Fig 1:**
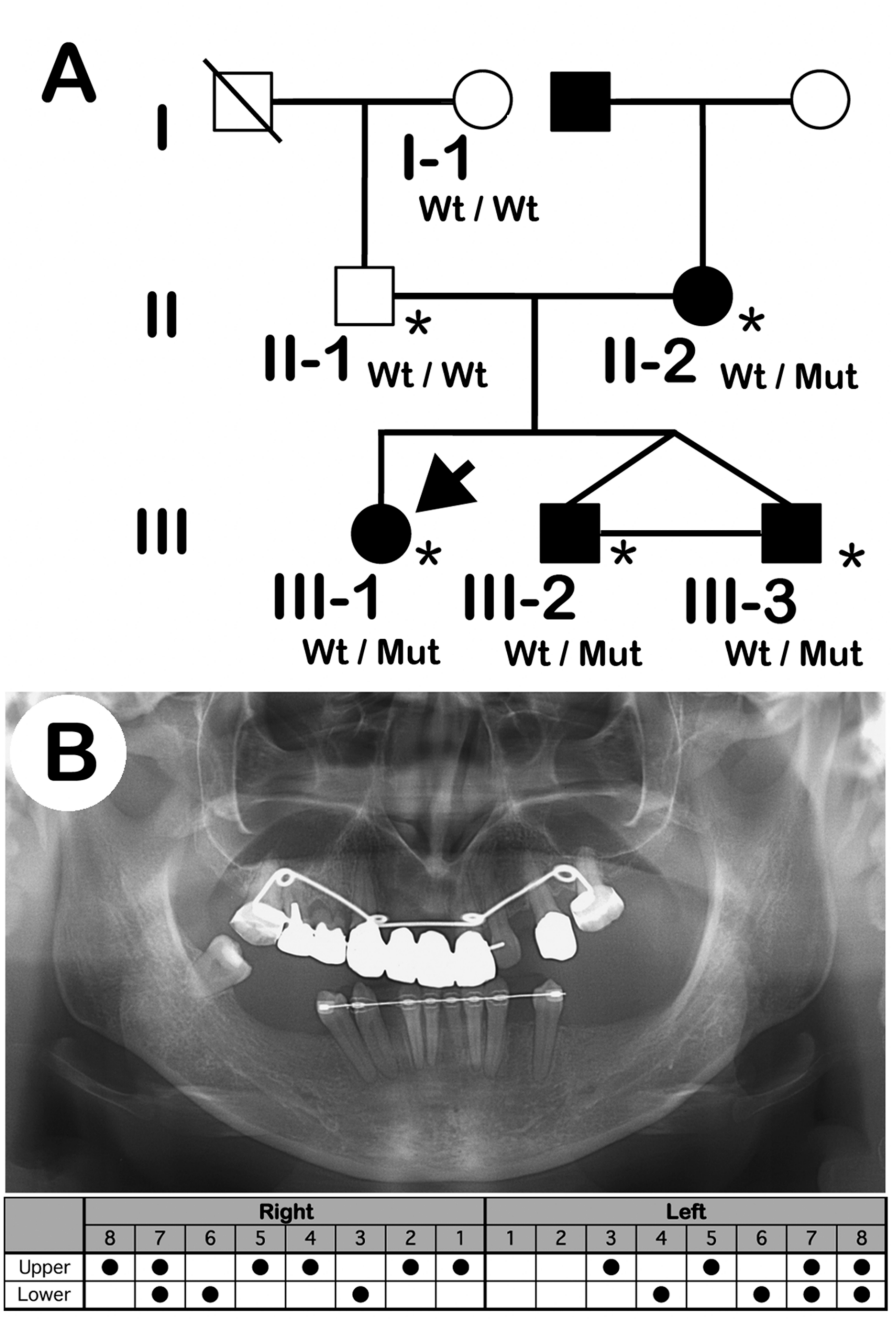
Pedigree and panoramic X-ray photograph. (A) Pedigree affected by nonsyndromic oligodontia. The pedigree displays an autosomal dominant mode of inheritance. Squares indicate males and circles indicate females. Black and white symbols indicate affected and unaffected individuals, respectively. The arrow indicates the proband, III-1. Asterisks indicate individuals analyzed by whole-exome sequencing. Mut, c.452-9G>A; wt, wild-type. (B) Panoramic X-ray (upper panel) and the missing tooth pattern (lower panel) of the proband (III-1).

WES was performed according to our previous report [28]. Briefly, four affected individuals (Fig 1A; II-2, III-1, III-2, and III-3) and one unaffected individual (II-1) from the family were analyzed. The resulting single-nucleotide variants were filtered based on the autosomal dominant inheritance model. Then, all intronic variants were analyzed by ESE finder (http://rulai.cshl.edu/cgi-bin/tools/ESE3/esefinder.cgi?process=home). Sanger sequencing confirmed the intronic nucleotide substitution in the *MSX1* gene of all available family members.

### RNA extraction from lymphoblastoid cell lines

Total RNA was extracted with TRIzol Reagent (Invitrogen Life Technologies, Carlsbad, CA) from an Epstein-Barr virus-transformed lymphoblastoid cell line generated from the proband blood. cDNA was then prepared in accordance with the manufacturer’s protocol (ReverTra Ace qPCR RT Master Mix, Toyobo, Tokyo, Japan). The cDNA fragment of *MSX1* was amplified with PCR, and sequenced on an ABI Prism 370 DNA Analyzer (Applied Biosystems).

### Minigene and cDNA of *MSX1* expression plasmids

The FLAG-tagged wild-type human *MSX1* cDNA (accession number: NM_002448) and W139X *MSX1* cloned into a pcDNA3 expression vector (Invitrogen, Grand Island, NY) have been previously described [20, 23]. The oligonucleotides of the *MSX1* gene, including the intronic region, were amplified with PCR using the following primers: forward, 5'-TGACTTCTTT GCCACTCGGT GTCAA-3', and reverse, 5'-AGCAGTGTGA GGGTTAAAGG GAAGG-3'. The template genomic DNA samples were isolated from the proband, III-1. The PCR conditions were 98°C for 1 min, followed by 35 cycles of 98°C for 10 sec, 55°C for 5 sec, and 72°C for 20 sec. To introduce the intronic region, the amplified DNA fragments were inserted into the FLAG-tagged *MSX1* cDNA expression vector.

### Immunolocalization of gene product of MSX1 minigene with intronic nucleotide substitution

To determine the subcellular localization of the product of the *MSX1* minigene with the intronic mutation, COS7 cells were transfected with the minigene or cDNA expression plasmids for the products using Lipofectamine 2000 (Invitrogen) as described previously [23]. Forty-eight hours post-transfection, the cells were fixed with 4% paraformaldehyde/Tris-buffered saline (TBS) and permeabilized with 1% Triton X-100/TBS prior to incubation with an anti-FLAG M2 monoclonal antibody (1:2000). The cells were then incubated with DAPI (1 mg/ml) and Cy3-conjugated goat anti-mouse antibody (1:1000; Jackson ImmunoResearch Laboratories Inc., West Grove, PA) in phosphate-buffered saline (PBS). After washing the cells with PBS three times, immunostaining signals were visualized under an Olympus BH-2 microscope.

### Sequencing analysis of cDNA generated from MSX1 minigene with intronic nucleotide substitution

To confirm an additional 7-bp insertion between exons 1 and 2, minigene-transfected COS7 cells were harvested and total RNA was isolated using TRIzol following the manufacturer’s protocol. Then, cDNA was synthesized by reverse transcription of 2 μg total RNA using random primers and the SuperScript VILO cDNA Synthesis Kit (Invitrogen).

### SDS-PAGE and western blotting

Western blotting was performed as described previously [29] with minor modifications. Briefly, COS7 transfectants were extracted with lysis buffer (1% Triton X-100, 1 mM EDTA in TBS, pH 7.5). Lysed products were centrifuged and the supernatant was used as the total lysate. For nuclear proteins, transfectant cells were suspended in hypotonic buffer (10 mM HEPES pH 7.5, 10 mM KCl, 1.5 mM MgCl_2_, 0.1 mM EDTA, 0.1% NP-40, protease inhibitors) and lysed by pipetting. Nuclei were separated by centrifugation at 1000 *g* for 15 min. The nuclear pellet was resuspended in extraction buffer (20 mM HEPES pH 7.5, 400 mM NaCl, 1.5 mM MgCl_2_, 0.1 mM EDTA, 10% glycerol, protease inhibitors) and incubated for 30 min on ice. The nuclear suspension was centrifuged and the supernatant was collected as the nuclear extract. These samples were subjected to 12% SDS-PAGE and transferred to Immobilon-P membranes (Millipore Corporation, Bedford, MA). The membranes were probed with an anti-FLAG M2 monoclonal antibody (1:2000; Sigma, St. Louis, MO), and signals on the blots were detected with a horseradish peroxidase-conjugated secondary antibody and ECL2 reagent (1:400; Amersham Life Science, Cleveland, OH).

## Results

### Clinical findings and family pedigree

Clinical examination, including radiographic analysis, confirmed the diagnosis of nonsyndromic oligodontia in the proband (III-1) and her two younger brothers (Fig 1A; III-2 and -3) with congenital absence of 11–17 permanent teeth (Table 1). The mother (II-2) presented with the mild missing tooth number phenotype (Table 1). All reported subjects had normal primary dentition, nails, skin, and hair. Of the six members studied, four were affected (two males and two females). The pedigree showed that the tooth agenesis mutation segregated in an autosomal dominant manner (Fig 1A).

**Table 1:** Missing teeth patterns of the affected members of the study family.

					Tooth phenotype															
ID	Gender	Age	# of missing teeth (*)		Right								Left							
					8	7	6	5	4	3	2	1	1	2	3	4	5	6	7	8
II-1	M	55	0	Upper																
				Lower																
II-2	F	54	4	Upper				●									●			
				Lower					●								●			
III-1	F	28	17(14)	Upper	●	●		●	●		●	●			●		●		●	●
				Lower		●	●			●						●		●	●	●
III-2	M	26	13(10)	Upper	●			●									●			
				Lower	●			●				●	●				●	●	●	●
III-3	M	26	11(7)	Upper	●			●									●			●
				Lower	●	●		●			●		●				●			●

*: Excluding 3rd molar.

●: Missing teeth.

### Mutation analysis

**Fig 2:**
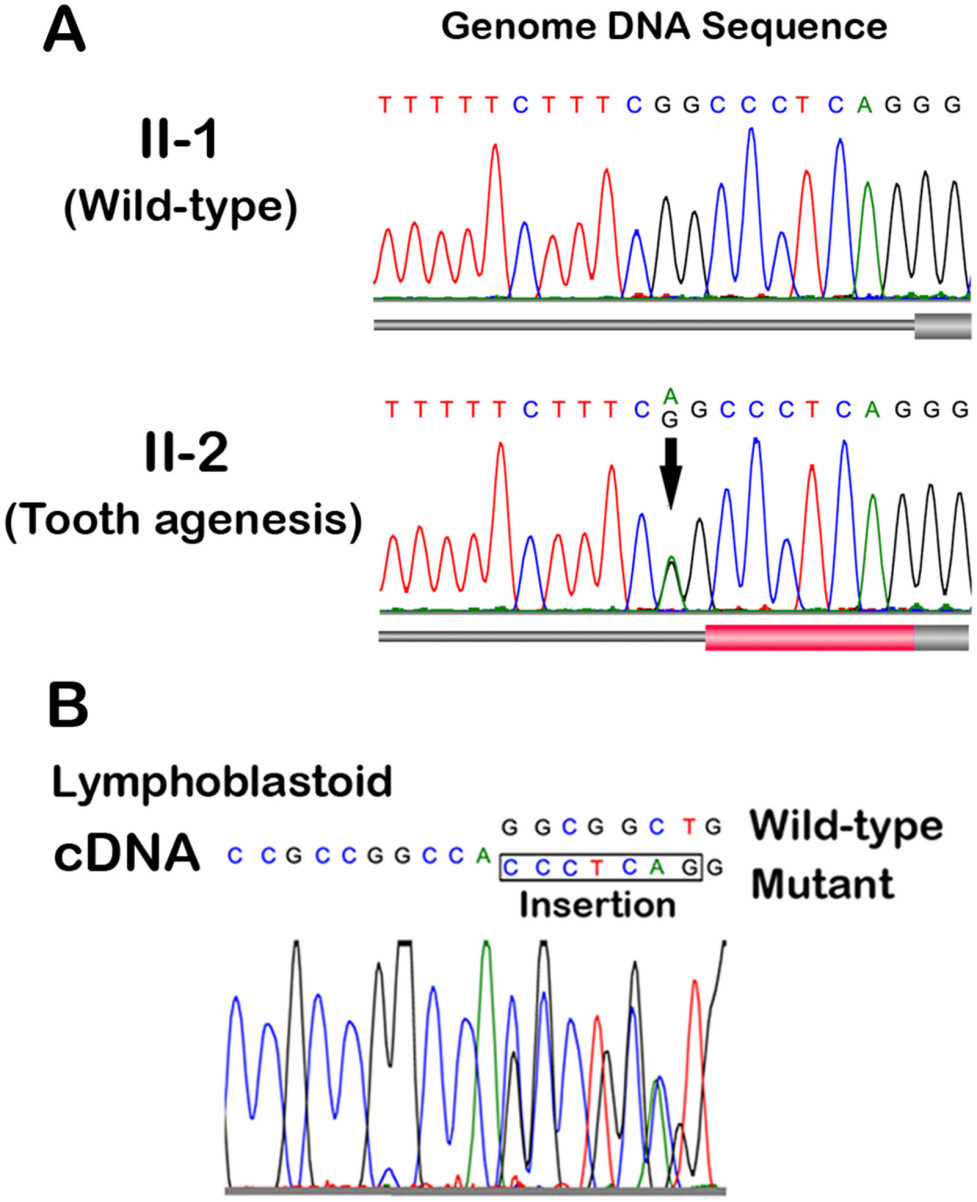
Nucleotide substitution in *MSX1* gene in a family with nonsyndromic tooth agenesis. (A) Genomic sequence analysis. Electropherograms of the junction between the intronic region and exon 2 of the *MSX1* gene. Unaffected (II-1) and affected (II-2) members of the family are indicated. There was a nucleotide substitution found in the intronic region nine nucleotides before the initiation site of the second exon (c.452-9G>A indicated by arrow). All of the affected members in the pedigree had the same heterozygous c.452-9G>A mutation. Thick cylinders, exons; red cylinder, predicted additional 7-nucleotide insertion. (B) cDNA sequence analysis. Electropherograms of the junction between exons 1 and 2 of the *MSX1* cDNA isolated from the lymphoblastoid cell lines generated from the proband (III-1). The 7-bp insertion is shown in the box.

To identify novel missense or loss of function variants shared by the affected individuals (Fig 1A; II-2 and III-1, -2, and -3), we performed WES of five members of the family, and identified 2,316 sequence alterations in the family. From the candidates, we selected variants shared among all affected individuals. Then, we excluded variants observed in the unaffected individuals, the synonymous variants, and variants in segmental duplications. Finally, all these variants registered in dbSNP137 or our in-house database (exome data of 575 Japanese individuals) were excluded. These steps ruled out all candidate genes except for 36 variants. After assessing these variants as likely causes of the dental condition, the most likely candidate was a novel heterozygous nucleotide substitution (c.452-9G>A) in the *MSX1* gene, since ESE-finder predicted that the nucleotide substitution would generate a novel splice acceptor site at 7bp before the original splice acceptor site for the second exon of the *MSX1* gene (c.451_452insCCCTCAG). The unaffected father (II-1) and grandfather (I-1) were not carrying this substitution (Fig 2A).

### Effect of the intronic nucleotide substitution on *MSX1* splicing

**Fig 3:**
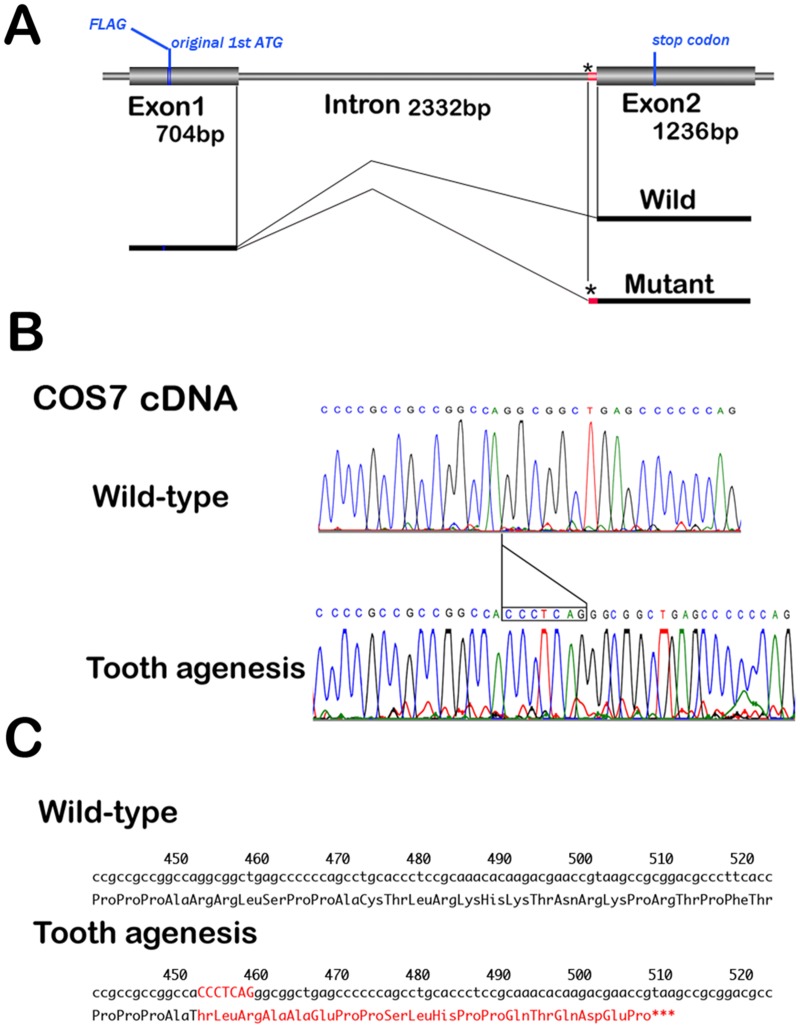
Sequence of cDNA generated by minigene. (A) Schematic diagram of the FLAG-tagged *MSX1* gene. Thick cylinders, exons; blue cylinder, FLAG-tag; asterisk, position at nucleotide substitution; red cylinder, 7-nucleotide insertion. (B) Electropherograms of the *MSX1* cDNA isolated from the COS7 cells transfected with *MSX1* minigene plasmids; wild-type (II-1) and c.452-9G>A (II-2). (C) Predicted amino acid sequences of wild-type (upper) and p.R151fsX20 (lower). The 7-bp insertion and 19 additional amino acids residues in the C-terminus are highlighted in red.

We investigated *MSX1* cDNA sequence isolated lymphoblastoid established from affected individual of the family. We observed heterozygosity for a 7-bp insertion at a position of the joint connecting two exons (Fig 2B). Then, to confirm that the insertion is generated by nucleotide substitution at c.452-9G>A in the *MSX1* gene, we constructed a minigene expression vector for *MSX1* with the c.452-9G>A variation (Fig 3A). Sequence analysis of the cDNA isolated from the minigene-transfected cells revealed that this nucleotide substitution mediates a 7bp-insertion between nucleotide positions 451 and 452 (Fig 3B). Therefore, the variant mRNA encodes a frame-shifted protein with a premature termination codon (p.R151fsX20; Fig 3C). Other than this mutation, no other heterozygous variants of known causative genes of tooth agenesis were identified.

### Subcellular localization and western blotting for MSX1 (c.452-9G>A)

**Fig 4:**
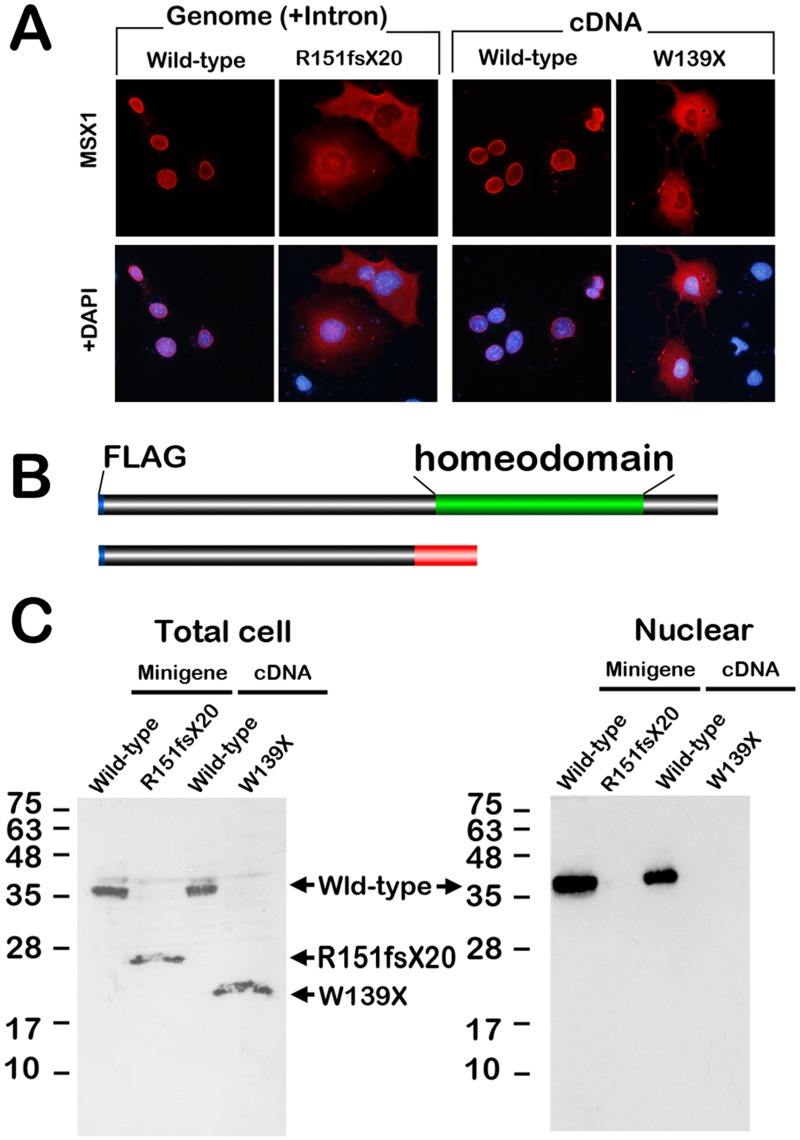
Characterization of the gene product of the *MSX1* gene with the c.452-9G>A substitution. (A) Immunolocalization of FLAG-tagged MSX1 protein in transfected COS7 cells. Nuclear translocation (wild-type) is disrupted by c.452-9G>A substitution in the intronic region (mutant). A diffuse signal is also observed in the transfectant of W139X MSX1, which is a C-terminal truncated mutant. (B) Schematic repetitions of wild and mutant MSX1 protein. The mutant MSX1 protein lacks the homeodomain (green cylinder in wild-type MSX1). Blue cylinder, FLAG tag; red cylinder, unrelated peptide generated by the insertion caused by the c.452-9G>A substitution. (C) Western blotting of cell lysate prepared from total cells (left) or nuclear fractions (right) of COS7 transfected with the *MSX1* minigene (FLAG tagged wild-type and c.452-9G>A) or cDNA (FLAG tagged wild-type and W139X) expression vectors. The molecular masses of the R151fsX20 and W139X MSX1 proteins are lower than that of wild-type MSX1.

The homeodomain is crucial for DNA binding, nuclear localization, and transcriptional activity [22, 23]. Because p.R151fsX20 lacks the domain (Fig 4B), we performed immunolocalization analysis of p.R151fsX20 MSX1 in the transfected COS7 cells. Whereas the significant nuclear localization of wild-type MSX1 was detected, that of p.R151fsX20 MSX1 was not observed in the gene-transfected cells in a manner similar to p.W139X MSX1 (Fig 4A), which we recently reported to be a novel tooth agenesis causative mutant of *MSX1* with a stop codon before the homeodomain [20]. Western blotting analysis indicated that the molecular mass of the mutant MSX1 was significantly lower than that of wild-type MSX1(Fig 4B and 4C). In addition, p.R151fsX20 and p.W139X MSX1 were not detected in the nuclear fraction of the transfected COS7 cells (Fig 4C). These results imply that the single nucleotide substitution interferes the mRNA splicing at the original splice acceptor site in the mutant genome.

## Discussion

### Intronic nucleotide substitution results in C-terminal truncation of MSX1

In a Japanese family with autosomal dominant nonsyndromic tooth agenesis (Fig 1A and 1B), we identified a novel splice mutation in the *MSX1* gene (Fig 2A). The heterozygous mutation (c.452-9G>A) generates a novel splice acceptor, resulting in a 7-nucleotide insertion in the cDNA at the first and second exon junction (Fig 3A and 3B). This intronic nucleotide substitution thus causes a frameshift mutation and generates a novel stop codon after an unrelated polypeptide sequence consisting of 19 amino acid residues (Fig 3C). The resultant mutant protein lacks the homeodomain (Fig 4B and 4C), which is crucial for nuclear localization of MSX1 and transcriptional regulation of target genes. We revealed disruption of the nuclear translocation potency of the truncated MSX1 generated by the c.452-9G>A substitution (Fig 4A). A single nucleotide substitution in the first intron of the *MSX1* gene has been reported previously as a possible causative variation [30]. In addition, a nonstop mutation of *MSX1* has been shown to be a cause of tooth agenesis. Nuclear translocation of the mutant MSX1 is impaired by the extra peptide consisting of 48 amino acid residues at the C-terminus [21]. These results also indicate that nuclear translocation of MSX1 is crucial for human tooth development. Our present observations show that the intronic nucleotide substitution we identified is the main cause of the tooth agenesis in our subject family.

### Genetic factors other than the *MSX1* mutation may influence tooth number

Strikingly, the missing tooth number phenotypes were found to be considerably different between the affected mother and her three children in our subject family (Fig 1A and Table 1). This observation suggests that some genetic risk factor(s) that can accelerate the missing tooth phenotype might have been inherited from the father, who shows normal tooth formation (Table 1). We have reported previously that a patient carrying the T174I MSX1 mutation, which causes nullity of transcriptional suppression activity, showed nine missing teeth, including four wisdom teeth, whereas other null mutations such as L205R MSX1 severely affected the formation of between 13 and 18 teeth [22]. The members of the affected family with W139X MSX1 also showed various numbers of missing teeth, from 7 to 18 [20]. In our current study, we report a mild phenotype involving the loss of four teeth caused by a haploinsufficiency of *MSX1*, which lacks the entire C-terminus structure including the homeodomain. This implies that loss of function in a single allelic *MSX1* could result in a much milder tooth phenotype and that particular genetic backgrounds may have influenced the number of missing teeth in all of the tooth agenesis cases that have been reported previously.

To identify the genetic factors that were transmitted from the father to the three children and are related to the phenotypic deterioration in our current study family, we attempted to analyze all of the exome sequence data of these family members. However, although several candidate genes were found, we were unable to identify specific gene mutations responsible for promoting the tooth phenotype in the pedigree. In addition, the missing tooth patterns were found to be slightly but significantly different between an identical twin pair of this family, strongly suggesting an effect of something other than genetic factors. Taken together, besides the genetic background, our present clinical and genetic observations clearly showed that epigenetic or environmental factors also affect the phenotype of missing tooth patterns in a patient with nonsyndromic tooth agenesis caused by *MSX1* haploinsufficiency. Further studies are required to clarify how to identify the gene mutations affect tooth numbers in cases with an *MSX1* haploinsufficiency.
